# Preoperative Hypoalbuminemia Predicts Infection, Fracture, and Repeat Revision After Revision Total Hip Arthroplasty; Prealbumin Stratification Does Not Refine Risk: A Retrospective Database Analysis

**DOI:** 10.3390/healthcare14070947

**Published:** 2026-04-04

**Authors:** Nicholas Reid Kiritsis, Alisa Diane Geier, Konstantinos Oikonomou, Jackson P. Midtlien, Isabel R. Shaffrey, John Shepherd Shields, Maxwell Kenneth Langfitt, Molly Amanda Hartzler

**Affiliations:** 1 School of Medicine, Wake Forest University, 1 Medical Center Blvd, Winston-Salem, NC 27157, USA; alisa.geier@gmail.com (A.D.G.); jackson.midtlien@wfusm.edu (J.P.M.); john.s.shields@advocatehealth.org (J.S.S.); maxwell.langfitt@advocatehealth.org (M.K.L.); molly.hartzler@advocatehealth.org (M.A.H.); 2 Department of Orthopaedic Surgery, School of Medicine, Wake Forest University, 1 Medical Center Blvd, Winston-Salem, NC 27157, USA; 3 School of Medicine, Virginia Commonwealth University, 1201 E Marshall St #4-100, Richmond, VA 23298, USA; oikonomouk@vcu.edu; 4 School of Medicine, Duke University, 2301 Erwin Rd, Durham, NC 27705, USA; isabelshaffrey@gmail.com

**Keywords:** albumin, hypoalbuminemia, revision hip arthroplasty, hip arthroplasty, complications, prealbumin, nutritional status, periprosthetic joint infection

## Abstract

**Highlights:**

**What are the main findings?**

- Preoperative hypoalbuminemia (<3.5 g/dL) is significantly associated with increased rates of surgical site infection, periprosthetic fracture, and repeat revision THA following rTHA, with elevated complication risk persisting for at least 5 years postoperatively.
- Prealbumin stratification among hypoalbuminemic rTHA patients did not differentiate complication risk at any follow-up interval, suggesting that short-term nutritional improvement may not reverse the long-term effects of chronic malnutrition.

**What are the implications of the main findings?**

- Preoperative serum albumin may benefit surgeons as a risk stratification tool in patients undergoing revision total hip arthroplasty to identify those at elevated risk for long-term complications.
- The limited utility of prealbumin in this setting suggests that last-minute nutritional resuscitation is unlikely to meaningfully reduce complication risk in chronically malnourished rTHA patients.

**Abstract:**

**Background/Objectives:** Hypoalbuminemia is a marker of poor nutritional status and has been associated with increased postoperative complications following total joint arthroplasty. However, its long-term implications in the revision total hip arthroplasty (THA) population are poorly characterized, and the utility of prealbumin to further risk-stratify these patients remains unclear. We aimed to study the association between preoperative hypoalbuminemia and complications after rTHA. **Methods:** We identified patients who underwent rTHA with preoperative albumin levels obtained within one month of surgery. Patients were divided into hypoalbuminemia (<3.5 g/dL) and normal albumin (3.5–6.0 g/dL) cohorts. A second analysis was conducted stratifying hypoalbuminemia patients by prealbumin level (<16 mg/dL vs. ≥16 mg/dL), measured within two weeks of surgery. Each cohort was 1:1 propensity score matched with a 1:1 nearest-neighbor greedy matching approach with a 0.10 standard deviation (SD) caliper, following a logistic regression to calculate patient propensity scores. Outcomes were compared at 90-day, 2-year, and 5-year intervals. **Results:** The matched cohorts included 4137 patients in both the hypoalbuminemia and normal-albumin groups. Hypoalbuminemia was significantly associated with increased short-term rates of any adverse event (38.9% vs. 22.5%; OR 2.195), wound dehiscence (5.4% vs. 3.1%; OR 1.808), surgical site infection (10.7% vs. 5.0%; OR: 2.271), and periprosthetic fracture (13.9% vs. 10.2%; OR: 1.414). Repeat revision THA was significantly more common within 90 days (6.6% vs. 4.5%; OR: 1.490). Periprosthetic fracture and prosthetic loosening were also more common within 2 years and 5 years (q = 0.001). There were no differences in repeat rTHA within 2 years and 5 years. Among hypoalbuminemic patients with prealbumin data, stratification by prealbumin level did not demonstrate any statistically significant differences in 90-day, 2-year, and 5-year complications. **Conclusions:** Hypoalbuminemia is a strong indicator of increased complication risk after rTHA, with increased risk for complications lasting to at least 5 years postoperatively. However, prealbumin stratification among hypoalbuminemic patients did not differentiate complication risk. These findings support preoperative albumin as a practical biomarker for risk stratification in rTHA patients.

## 1. Introduction

Osteoarthritis is a progressively devastating disease, with hip and knee osteoarthritis ranking as the 11th primary contributor to disability globally [1,2,3]. Total hip arthroplasty is a highly effective treatment for advanced-stage osteoarthritis. By 2030, the number of primary total hip arthroplasties and with this revision total hip arthroplasties performed is expected to increase by 174% and 137% respectively [4]. Revision surgeries, characterized by increased duration and complexity, are associated with a higher risk and incidence of complications compared to primary procedures [5,6,7]. Significant emphasis has been placed on presurgical optimization strategies to identify and manage modifiable risk factors in order to lower the likelihood of adverse events and improve overall patient outcomes [8].

Prior studies have investigated preoperative albumin as an important marker of nutritional status and a preoperative risk factor; however, there has been minimal investigation into the predictive value of prealbumin levels in the context of chronically hypoalbuminemic patients as a marker of short-term supplementation. Malnutrition, characterized by albumin <3.5 g/dL, can greatly hinder wound healing, increase risk of infection, complications, reoperations and overall mortality [9,10,11,12,13]. Although albumin and prealbumin are both used to evaluate nutritional status, prealbumin may serve as more sensitive marker for detecting acute nutritional changes, owing to its shorter half-life of 2–3 days [14]. However, its utility in preoperative optimization and in predicting short-term and long-term complications in revision total hip arthroplasty has yet to be established. In modern practice, both albumin and prealbumin are frequently ordered as part of preoperative nutritional screening panels, reflecting either default order sets or some level of uncertainty about which marker best characterizes perioperative risk. Additional, unused lab values add direct expense and provide an unknown benefit if lacking clinical relevance. If prealbumin does not meaningfully refine risk stratification beyond the known value of albumin levels, its routine preoperative measurement in rTHA patients would be add a futile burden on patients and the healthcare system. Conversely, if prealbumin identifies a subset of hypoalbuminemic patients at lower risk of complications, it could serve as a useful tool to guide surgical timing. To our literature review, no large-scale study has examined whether prealbumin stratification adds predictive value over albumin alone in rTHA patients.

In this study, we aimed to evaluate (1) whether preoperative hypoalbuminemia was associated with increased rates of surgical site infection, periprosthetic joint infection, periprosthetic fracture, and revision total hip arthroplasty at 90-day, 2-year, and 5-year intervals after rTHA, and (2) whether stratifying hypoalbuminemic patients by preoperative prealbumin level (<16 vs. ≥16 mg/dL) was associated with any differences in complication rates, and we hypothesized that preoperative hypoalbuminemia would be independently associated with increased rates of infection, fracture, and repeat rTHA and that prealbumin would further stratify risk within the hypoalbuminemic cohort.

## 2. Materials and Methods

This was a retrospective cohort study using TriNetX (Cambridge, MA), a research database offering access to deidentified patient records compiled from healthcare organizations. The analysis was conducted using the Research network, which included 104 healthcare organizations across the United States, including academic medical centers and large health systems. All data are fully de-identified in accordance with the Health Insurance Portability and Accountability Act (HIPAA) Safe Harbor method, and this study was therefore exempt from institutional review board review.

We performed two analyses on patients who underwent revision total hip arthroplasty (rTHA). Patients within the database were identified using Current Procedural Terminology (CPT) codes 27134, 27137, and 27138. The first analysis compared individuals who underwent rTHA with low preoperative albumin levels to those with normal albumin levels. Low albumin was defined as an albumin level within 1 month of surgery between 0.0–3.49 g/dL, and normal albumin was defined by a value of 3.5–6.0 g/dL. rTHA patients without an albumin level prior to surgery were excluded from the analysis, and if patients had multiple values in their record, the result closest to surgery was used. The second analysis was to evaluate outcomes among the hypoalbuminemic cohort stratified by their prealbumin status. We compared those with low prealbumin (<16 mg/dL) to those with normal prealbumin (≥16 mg/dL), measured within the two weeks before surgery.

To identify surgical indications for rTHA, we queried for common International Classification of Diseases, Tenth Edition (ICD-10) diagnoses before surgery. The investigated indications included infection and inflammatory reaction due to internal joint prosthesis (T84.51, T84.52), mechanical loosening of the internal right or left hip prosthetic joint (T84.030, T84.031), periprosthetic fracture around the internal prosthetic hip joint (M97.0), recurrent dislocation of the hip (M24.45), and other forms of hip instability (M25.35), as shown in Table 1.

The cohorts in both analyses were propensity score matched with a 1:1 nearest-neighbor greedy matching approach with a 0.10 standard deviation (SD) caliper, following a logistic regression to calculate patient propensity scores. The matching parameters included age, sex, race, body mass index (BMI), type 2 diabetes mellitus, nicotine dependence, chronic ischemic heart disease, heart failure, chronic kidney disease, and chronic obstructive pulmonary disease.

Postoperative outcomes were evaluated within 90 days, 2 years, and 5 years of surgery. The primary outcomes for both analyses included 90-day wound dehiscence and SSI, along with periprosthetic fracture and subsequent revision THA at all time points. We also queried for additional complications including any adverse event (AAE), pulmonary embolism (PE), deep vein thrombosis (DVT), myocardial infarction (MI), cerebral infarction, sepsis, blood transfusion, hematoma, emergency department (ED) visits, inpatient admissions, prosthetic loosening, and venous thromboembolism (VTE).

The baseline demographics and comorbidities of the two cohorts were assessed using *t*-tests and chi-squared tests (Table 2). Statistical analyses of baseline demographics and postoperative outcomes were conducted within TriNetX, and outcomes are presented as rates and odds ratios (ORs) with *p*-values, *q*-values and 95% confidence intervals (CIs). A Benjamini–Hochberg (BH) false discovery rate (FDR) correction was applied across all comparisons in Table 3, Table 4, Table 5 and Table 6 to reduce the risk of type I error. This method was selected over Bonferroni correction given the exploratory nature of the study and the expected correlation between clinical outcomes. Statistical significance was defined as a BH-adjusted q-value < 0.05. Both original *p*-values and adjusted q-values are reported in all outcome tables.

There were 4264 patients in the low albumin cohort and 10,408 patients in the normal albumin cohort who underwent revision total hip arthroplasty (rTHA). The 1:1 propensity score matching yielded 4137 patients per cohort. Prior to matching, the hypoalbuminemic group was older (70.7 ± 12.9 vs. 66.3 ± 12.8 years; *p* < 0.001) and had higher rates of comorbidities, including type 2 diabetes, heart failure, chronic kidney disease, and COPD (all *p* < 0.001). After matching, all variables were well-balanced with *p* > 0.05 and SMD < 0.05, except for BMI (28.8 vs. 29.2; *p* = 0.006), which remained higher in the normal-albumin group (Table 2).

## 3. Results

### 3.1. Ninety-Day Outcomes

Within 90 days of surgery, all primary outcomes favored the normal albumin cohort. Hypoalbuminemia was associated with higher rates of SSI (10.7% vs. 5.0%, q = 0.001), wound dehiscence (5.4% vs. 3.1%, q = 0.001), periprosthetic fracture (13.9% vs. 10.2%, q = 0.001), and revision THA (6.6% vs. 4.5%, q = 0.001). Secondary outcomes, including any adverse event (38.9% vs. 22.5%, q = 0.001) were elevated in the low-albumin group (Table 3).

### 3.2. Two-Year Outcomes

Within 2 years, low albumin remained associated with higher complication rates. Rates of a periprosthetic fracture (16.1% vs. 12.7%, q = 0.001) remained significantly higher in the hypoalbuminemic cohort. There was no longer a significant difference in the rate of subsequent revision hip arthroplasty (9.6% vs. 8.6%, q = 0.201). Rates of periprosthetic loosening (4.2% vs. 6.6%, q = 0.001) favored the low-albumin group (Table 4).

### 3.3. Five-Year Outcomes

At 5 years postoperative, low albumin patients continued to have greater complications. Their rates of periprosthetic fracture continued to differ from matched controls (17.0% vs. 13.7%, q = 0.001); however, rates of repeat rTHA were not significantly different (10.0% vs. 9.6%, *p* = 0.554). Rates of loosening continued to favor the hypoalbuminemic cohort (Table 5).

### 3.4. Prealbumin-Stratified Outcomes in Hypoalbuminemic Patients

Among patients with hypoalbuminemia, stratification by prealbumin level (<16 mg/dL vs. ≥16 mg/dL, measured within two weeks prior to surgery) revealed no statistically significant differences in primary or secondary complication rates at 90 days, 2 years, or 5 years per q-values greater than 0.05.

At 90 days, rates of all four primary outcomes were similar between groups. Surgical site infection occurred in 14.3% of patients with low prealbumin and 12.9% with normal prealbumin (q = 0.900), while wound dehiscence was reported in 10.0% vs. 7.1% (q = 0.531). A subsequent revision THA occurred in 12.1% vs. 10.7% (q = 0.900). Among secondary outcomes, rates of any adverse event (50.0% vs. 41.4%, q = 0.268) and periprosthetic hip fracture (15.0% vs. 19.3%, q = 0.188) were also not statistically different.

Within 2 years, our primary outcomes again showed no differences. Subsequent revision THA was needed in 22.1% of those with low prealbumin vs. 17.1% in the control group (q = 0.437). Rates of periprosthetic fracture (18.6% vs. 25.0%, q = 0.333) remained equivalent.

At 5 years, there were still no significant differences in primary outcomes. The incidence of revision THA was 10.0% vs. 8.6% (q = 0.729). Other outcomes also remained comparable, with rates of periprosthetic fracture showing no statistically significant difference (Table 6).

## 4. Discussion

To our literature review, this is the largest study on serum albumin in revision hip arthroplasty. In this large-scale, retrospective review of the TriNetX database, we found that preoperative hypoalbuminemia was significantly associated with adverse events in a representative rTHA population [15]. Patients with a low preoperative serum albumin level experienced increased rates of SSI, periprosthetic fracture, implant loosening, and need for another revision hip arthroplasty. Of note, many of these associations were present at both short-term and long-term follow up intervals, which underlies the durability of perioperative albumin levels as a predictor of outcomes beyond the initial postoperative period. Additionally, a sub-analysis of the hypoalbuminemia cohort demonstrated that a normal prealbumin level, indicating a better short-term nutrition status, was not associated with improved outcomes. Our findings support albumin as a practical marker for risk stratification in the rTHA population; however, it is important to acknowledge that only prospective studies can determine whether preoperative nutritional optimization translates to improved outcomes in this population.

Published research has consistently demonstrated an association between low albumin and increased complications, costs, and health care utilization after primary and revision arthroplasty, with some evidence showing that a level <3.5 g/dL has a strong influence on infection, readmission, overall complications, and mortality after THA [9,10,11,12,13]. Our results did not demonstrate the same extent of elevated infection risk previously seen, which showed that hypoalbuminemic patients were at a 3.15-times greater odds of infection within 30 days, compared to our 90-day odds of 2.27 (SSI) [10]. We also failed to see the same disparity in rates of venous thromboembolism as Heimroth et al. in their analysis of malnourished patients in the ACS-NSQIP database who underwent rTHA, with our data showing a near 50% increase in 90-day risk compared to their findings of a more than double 30-day risk of DVT or PE within 30 days [16]. Direct comparison is limited by the difference in follow-up windows, and the higher baseline event rates accumulated over 90 days in both our hypoalbuminemic and normal albumin cohorts likely contribute to the more conservative odds ratios we observed relative to 30-day studies.

Rates of revision arthroplasty only significantly differed in the 90-day postoperative window, which aligns with a study of the ACS-NSQIP database by Newman et al. that showed a 97% increased risk of reoperation within 30 days for hypoalbuminemic patients [9]. Similar to our findings, a recent meta-analysis of over 50,000 patients found a 93% increased risk of all-cause complications compared to patients with normal albumin levels [17]. In another analysis of the American College of Surgeons National Surgical Quality Improvement Program (ACS-NSQIP) database looking at complications after revision hip, knee, and shoulder arthroplasty, hypoalbuminemia conferred a 4.32-times risk of wound infection and 2.78-times risk of deep surgical site infection within 30 days [18]. We found hypoalbuminemia to confer a 1.85-times increased odds of readmission within 90 days, which aligns with prior data from Khoshbin et al. Their research indicated that hip arthroplasty patients with hypoalbuminemia have a significantly higher risk of readmission, with a 2.01-fold increased odds within 30 days of surgery [19].

Albumin is a strong indicator of nutritional status and postoperative risk, with greater prediction value than elevated BMI, tobacco use, diabetes, anemia, poor kidney function, and hyponatremia in primary THA [19]. In the era of increasing healthcare costs and resource scarcity, there should remain an emphasis on indications for risk stratification. Albumin levels have consistently demonstrated their utility at predicting adverse outcomes, but there remains debate on the utility of screening every patient. In a review of 819 primary joint arthroplasty patients, Rao et al. investigated the prevalence of abnormal nutritional values including albumin <3.5 g/dL, transferrin <200 mg/dL, and total lymphocyte count <1.5 cells/µL^3^ [20]. They found abnormal values for albumin in 2.6%, transferrin in 5.6%, and total lymphocyte count in 25%. However, only 1.7% of patients had two or more abnormal values, showing little overlap between the tests. This primary total hip arthroplasty population significantly differs from that of rTHA, where the comorbidity load is greater and 10–20% of patients present with abnormal albumin levels [12,21]. Additionally, hypoalbuminemia has been shown to predict a 16.2% increase in index hospitalization costs alone, without considering the expenses and sequelae of more frequent postoperative complications [11,22]. Nearly half of the patients presented with an infected prosthesis, which is associated with a nearly six-fold risk of general reoperation, likely due to two-stage exchanges with an antibiotic spacer. As of 2023, revisions for infection also cost over $11,000 more than aseptic revisions [15,23,24].

Conversely, several studies raise important nuances regarding screening thresholds and the predictive value of prealbumin. While many studies use 3.5 g/dL as the threshold for hypoalbuminemia, Nelson et al. suggested risk increases may begin at lower levels (3.0 g/dL) in total hip arthroplasty, while Black et al. proposed a threshold closer to 3.94 g/dL may be more sensitive and specific in joint arthroplasty as a whole [25,26]. These differing thresholds highlight an important limitation of the present study. By using the conventional 3.5 g/dL cutoff, our cohort likely includes patients with mild hypoalbuminemia (3.0–3.49 g/dL) alongside those with more severe deficits, potentially minimizing the observed differences if postoperative risk is concentrated in severely malnourished patients. If the ideal inflection point is closer to 3.94 g/dL as suggested by Black et al., a meaningful proportion of patients in our normal albumin cohort may have been misclassified, biasing our odds ratios toward 1.0.

An important consideration when interpreting our findings is that in addition to being a marker of nutrition, serum albumin is a negative acute-phase reactant whose reduction can reflect systemic inflammatory states, hepatic dysfunction, or systemic illness severity. In our matched cohort, approximately 42% of hypoalbuminemic patients presented with an infected prosthesis as a surgical indication, which may suppress albumin levels independent of that patient’s nutritional status. This introduces the possibility that hypoalbuminemia in a substantial proportion of our cohort may represent a marker of systemic disease and physiologic stress rather than a modifiable nutritional deficit amenable to preoperative intervention.

Regarding prealbumin, our findings should be interpreted in the context of chronically hypoalbuminemic patients undergoing rTHA, rather than as a statement about prealbumin’s clinical utility. Our data showed that hypoalbuminemic patients with normalized prealbumin had near-equivalent outcomes to those with low prealbumin, with no differences noted after Benjamini–Hochberg correction at all follow-up intervals. This finding supports the interpretation that chronic malnutrition, as reflected by serum albumin, is the primary driver of long-term complication risk in this population and that short-term nutritional recovery sufficient to normalize prealbumin does not reverse that underlying risk. In the isolated setting of patients already identified as hypoalbuminemic prior to rTHA, our data suggest that prealbumin does not further stratify complication risk. The chronic nutritional deficit reflected by albumin represents a more durable indicator than the acute nutritional status captured by prealbumin. Prealbumin has demonstrated value in predicting complications in elective spine surgery, where low levels have been associated with higher infection risk [27]. The findings in this study are more attributable to the clinical context rather than an inherent limitation of prealbumin as a biomarker.

Many studies on perioperative lab values in primary and revision total hip arthroplasty are limited by their size, and large-scale studies by their follow-up period, as some databases such as the American College of Surgeons National Surgical Quality Improvement Program only follow patients for 30 days following surgery [18,21]. Additionally, many other studies have shown more severe disparities in complication rates between patients with low and normal albumin, which may speak to the thorough propensity score matching present in this analysis.

## 5. Limitations and Conclusions

This study has several limitations that should be considered when interpreting the findings, many of which are inherent to database research. First, the TriNetX database has an inherent selection bias, as the aggregated data is primarily sourced from academic health centers and large health systems. Community hospitals, rural facilities, and independent surgery centers are likely underrepresented, and the findings may not accurately assess outcomes in these populations that may be less likely to receive an extensive preoperative workup.

Second, this analysis is entirely reliant on electronic medical record coding accuracy and recording bias. As ICD-10 diagnosis codes and CPT procedure codes are primarily used for billing, it is possible that inaccuracies have mischaracterized patient characteristics or outcomes. Any complications managed outside of the included health systems and care sites or patients that do not present for follow-up are not captured within the database, which may underestimate complication rates. Conversely, we believe patients receiving an rTHA are likely to return to their index surgeon for management of any complications, which would negate any lost adverse events.

Third, the database does not capture many clinically relevant details known to influence patient outcomes after rTHA, including implant type, surgical approach, bone quality, surgeon volume, and surgeon experience. Other variables and outcome measures like patient functional status, frailty, and patient-reported outcomes are similarly not available for consideration. Likewise, the employed propensity score matching methods aid in reducing confounding by the included covariates, but it does not account for other unmeasured or unavailable factors. Each patient’s complexity goes beyond their chart and diagnosis codes. We were also limited with the sample sizes in our analyses, especially in the prealbumin comparison that only included 140 patients per group.

Finally, this study was retrospective and observational in nature, which prevents us from claiming causality. While our findings support preoperative albumin as a prognostic marker in rTHA, we cannot establish from these data that hypoalbuminemia directly caused the observed complications. We also lack any evidence to claim that correcting albumin levels will reduce complication rates. Furthermore, it is possible that the conventional 3.5 g/dL cutoff misclassified patients at either end of the hypoalbuminemia spectrum.

In conclusion, hypoalbuminemia is a robust predictor of complications after rTHA, with increased risk for complications lasting to at least 5 years postoperatively. Prealbumin stratification among hypoalbuminemic patients did not differentiate complication risk, suggesting that short-term nutritional improvement may not reverse the long-term effects of chronic malnutrition. These findings support preoperative albumin as a practical biomarker for risk stratification in rTHA patients and highlight the need for proactive nutritional assessment before revision arthroplasty.

## Figures and Tables

**Table 1:** Preoperative diagnoses and likely surgical indications before and after matching for hypoalbuminemia versus normal albumin patients undergoing revision total hip arthroplasty.

	Albumin 0–3.49 (Before Matching, *n* = 4264)	Albumin 3.5–6 (Before Matching, *n* = 10,408)	*p*-Value (Before Matching)	Albumin 0–3.49 (After Matching, *n* = 4137	Albumin 3.5–6 (After Matching, *n* = 4137)	*p*-Value (After Matching)
Infection or inflammatory reaction to internal joint prosthesis (*n*, %)	1859 (43.6%)	2696 (25.9%)	<0.001 *	1745 (42.2%)	1771 (42.8%)	0.587
Mechanical loosening (*n*, %)	750 (17.6%)	2622 (25.2%)	<0.001 *	749 (18.1%)	720 (17.4%)	0.406
Periprosthetic fracture (*n*, %)	1245 (29.2%)	1810 (17.4%)	<0.001 *	1175 (28.4%)	1204 (29.1%)	0.473
Recurrent hip dislocation (*n*, %)	222 (5.2%)	499 (4.8%)	0.346	211 (5.1%)	210 (5.1%)	1.000
Hip instability (*n*, %)	127 (3.1%)	500 (4.8%)	<0.001 *	128 (3.1%)	120 (2.9%)	0.644
Other or Unknown	61 (1.3%)	2281 (21.9%)	<0.001 *	129 (3.1%)	112 (2.7%)	0.296

* Denotes statistical significance *p* < 0.05.

**Table 2:** Baseline patient characteristics before and after propensity score matching.

	Albumin 0–3.49 (Before Matching, *n* = 4264)	Albumin 3.5–6 (Before Matching, *n* = 10,408)	*p*-Value	Albumin 0–3.49 (After Matching, *n* = 4137)	Albumin 3.5–6 (After Matching, *n* = 4137)	*p*-Value	Standardized Mean Difference (SMD)
Age at Index (mean ± SD)	70.7 ± 12.9	66.3 ± 12.8	<0.001 *	70.5 ± 12.9	70.6 ± 12.3	0.769	0.006
BMI (mean ± SD)	28.8 ± 7.4	29.7 ± 6.7	<0.001 *	28.8 ± 7.4	29.2 ± 6.7	0.006 *	0.069
Male (*n*, %)	1702 (40.3%)	4423 (43.1%)	0.002 *	1669 (40.3%)	1695 (41.0%)	0.561	0.013
White (*n*, %)	3400 (80.5%)	8262 (80.6%)	0.982	3336 (80.6%)	3396 (82.1%)	0.090	0.037
Black or African American (*n*, %)	370 (8.8%)	958 (9.3%)	0.275	363 (8.8%)	348 (8.4%)	0.556	0.013
Asian (*n*, %)	36 (0.9%)	105 (1.0%)	0.341	34 (0.8%)	31 (0.7%)	0.709	0.008
Type 2 Diabetes Mellitus (*n*, %)	1299 (30.8%)	2238 (21.8%)	<0.001 *	1250 (30.2%)	1268 (30.7%)	0.667	0.009
Nicotine Dependence (*n*, %)	938 (22.2%)	1943 (18.9%)	<0.001 *	900 (21.8%)	953 (23.0%)	0.162	0.031
Chronic Ischemic Heart Disease (%)	1355 (32.1%)	2186 (21.3%)	<0.001 *	1293 (31.3%)	1338 (32.3%)	0.288	0.023
Heart Failure (*n*, %)	1121 (26.6%)	1253 (12.2%)	<0.001 *	1040 (25.1%)	1001 (24.2%)	0.320	0.022
Chronic Kidney Disease (CKD) (*n*, %)	1143 (27.1%)	1592 (15.5%)	<0.001 *	1073 (25.9%)	1051 (25.4%)	0.580	0.012
Chronic Obstructive Pulmonary Disease (COPD) (*n*, %)	913 (21.6%)	1286 (12.5%)	<0.001 *	864 (20.9%)	883 (21.3%)	0.609	0.011

* Denotes statistical significance *p* < 0.05.

**Table 3:** 90-day complication rates, *p*-values, *q*-values, and odds ratios for hypoalbuminemic vs. normal albumin patients undergoing revision total hip arthroplasty (*n* = 4137).

	^a^ Hypoalbuminemia Cohort (*n*, %)	^b^ Normal Albumin Cohort (*n*, %)	*p*-Value	*q*-Value	Odds Ratio (95% CI)
**^†^** Any Adverse Event (*n*, %)	1609, 38.9%	930, 22.5%	<0.001	0.001 *	2.195 * (1.994, 2.416)
Dehiscence (*n*, %)	224, 5.4%	127, 3.1%	<0.001	0.001 *	1.808 * (1.447, 2.257)
Transfusion (*n*, %)	553, 13.4%	283, 6.8%	<0.001	0.001 *	2.101 * (1.808, 2.442)
Acute MI (*n*, %)	123, 3.0%	95, 2.3%	0.055	0.115	1.304 (0.994, 1.710)
Cerebral Infarct (*n*, %)	108, 2.6%	75, 1.8%	0.014	0.032 *	1.452 * (1.078, 1.955)
Pulmonary Embolism (PE) (*n*, %)	143, 3.5%	97, 2.3%	0.003	0.008 *	1.491 * (1.148, 1.937)
Deep Vein Thrombosis (DVT) (*n*, %)	162, 3.9%	113, 2.7%	0.003	0.008 *	1.451 * (1.137, 1.853)
Venous Thromboembolism (DVT or PE) (*n*, %)	280, 6.8%	190, 4.6%	<0.001	0.001 *	1.508 * (1.248, 1.823)
Surgical Site Infection (SSI) (*n*, %)	444, 10.7%	208, 5.0%	<0.001	0.001 *	2.271 * (1.915, 2.694)
Emergency Department Visit (*n*, %)	981, 23.7%	758, 18.3%	<0.001	0.001 *	1.386 * (1.246, 1.541)
Hematoma (*n*, %)	55, 1.3%	31, 0.7%	0.009	0.021 *	1.785 * (1.147, 2.777)
Sepsis (*n*, %)	415, 10.0%	182, 4.4%	<0.001	0.001 *	2.423 * (2.024, 2.901)
Inpatient Admission (*n*, %)	1712, 41.4%	1141, 27.6%	<0.001	0.001 *	1.854 * (1.691, 2.033)
Hip Periprosthetic Fracture (*n*, %)	575, 13.9%	424, 10.2%	<0.001	0.001 *	1.414 * (1.237, 1.616)
Hip Prosthetic Loosening (*n*, %)	126, 3.0%	214, 5.2%	<0.001	0.001 *	0.576 * (0.460, 0.721)
Revision THA (*n*, %)	274, 6.6%	188, 4.5%	<0.001	0.001 *	1.490 * (1.231, 1.803)

^a^ Low albumin was defined as <3.5 g/dL. ^b^ Normal albumin was defined as 3.5–6.0 g/dL. **^†^** Any adverse event: dehiscence, DVT, PE, MI, cerebral infarct, transfusion, hematoma, SSI, sepsis. * Denotes statistical significance *q* < 0.05.

**Table 4:** Two-year complication rates, *p*-values, and odds ratios for hypoalbuminemic vs. normal albumin patients undergoing revision total hip arthroplasty (*n* = 4137).

	^a^ Hypoalbuminemia Cohort (*n*, %)	^b^ Normal Albumin Cohort (*n*, %)	*p*-Value	*q*-Value	Odds Ratio (95% CI)
Hip Periprosthetic Fracture (*n*, %)	667, 16.1%	525, 12.7%	<0.001	0.001 *	1.322 * (1.169, 1.496)
Hip Prosthetic Loosening (*n*, %)	175, 4.2%	273, 6.6%	<0.001	0.001 *	0.625 * (0.514, 0.760)
Revision THA (*n*, %)	396, 9.6%	354, 8.6%	0.108	0.201	1.131 (0.973, 1.315)

^a^ Low albumin was defined as <3.5 g/dL. ^b^ Normal albumin was defined as 3.5–6.0 g/dL. * Denotes statistical significance *q* < 0.05.

**Table 5:** Five-year complication rates, *p*-values, and odds ratios for hypoalbuminemic vs. normal albumin patients undergoing revision total hip arthroplasty (*n* = 4137).

	^a^ Hypoalbuminemia Cohort (*n*, %)	^b^ Normal Albumin Cohort (*n*, %)	*p*-Value	*q*-Value	Odds Ratio (95% CI)
Hip Periprosthetic Fracture (*n*, %)	702, 17.0%	566, 13.7%	<0.001	0.001 *	1.289 * (1.143, 1.454)
Hip Prosthetic Loosening (*n*, %)	199, 4.8%	303, 7.3%	<0.001	0.001 *	0.639 * (0.532, 0.769)
Revision THA (*n*, %)	413, 10.0%	397, 9.6%	0.554	0.201	1.045 (0.904, 1.208)

^a^ Low albumin was defined as <3.5 g/dL. ^b^ Normal albumin was defined as 3.5–6.0 g/dL. * Denotes statistical significance *q* < 0.05.

**Table 6:** Complication rates, *p*-values, and *q*-values for patients with low vs. normal prealbumin (*n* = 140 each) within two weeks of surgery in hypoalbuminemic (<3.5 g/dL) patients undergoing revision total hip arthroplasty at 90-day, 2-year, and 5-year timepoints.

	90 Days			2 Years			5 Years		
	^a^ Low Prealbumin (*n*, %)	^b^ Normal Prealbumin (*n*, %)	*p*-Value; *q*-Value	^a^ Low Prealbumin (*n*, %)	^b^ Normal Prealbumin (*n*, %)	*p*-Value; *q*-Value	^a^ Low Prealbumin (*n*, %)	^b^ Normal Prealbumin (*n*, %)	*p*-Value; *q*-Value
**^†^** Any Adverse Event (*n*, %)	70 (50.0%)	58 (41.4%)	0.150; 0.268	–	–	–	–	–	–
Dehiscence (*n*, %)	14 (10.0%)	10 (7.1%)	0.393; 0.531	–	–	–	–	–	–
Transfusion (*n*, %)	22 (15.7%)	20 (14.3%)	0.738; 0.900	–	–	–	–	–	–
Venous Thromboembolism (DVT or PE) (*n*, %)	15 (10.7%)	10 (7.1%)	0.295; 0.437	–	–	–	–	–	–
Surgical Site Infection (*n*, %)	20 (14.3%)	18 (12.9%)	0.727; 0.900	–	–	–	–	–	–
Sepsis (*n*, %)	23 (16.4%)	17 (12.1%)	0.306; 0.437	–	–	–	–	–	–
Inpatient Admission (*n*, %)	83 (59.3%)	66 (47.1%)	0.042; 0.091	–	–	–	–	–	–
Hip Periprosthetic Fracture (*n*, %)	21 (15.0%)	27 (19.3%)	0.341; 0.474	26 (18.6%)	35 (25.0%)	0.193; 0.333	28 (20.0%)	40 (28.6%)	0.094; 0.188
Hip Prosthetic Loosening (*n*, %)	10 (7.1%)	10 (7.1%)	1.000; 1.000	10 (7.1%)	10 (7.1%)	1.000; 1.000	10 (7.1%)	10 (7.1%)	1.000; 1.000
Revision THA (*n*, %)	17 (12.1%)	15 (10.7%)	0.707; 0.900	31 (22.1%)	24 (17.1%)	0.292; 0.437	32 (22.9%)	25 (17.9%)	0.299; 0.729

^a^ Low prealbumin was defined as <16 mg/dL. ^b^ Normal prealbumin was defined as ≥16 mg/dL. **^†^** Any adverse event: dehiscence, DVT, PE, MI, cerebral infarct, transfusion, hematoma, SSI, sepsis.

## Data Availability

The data analyzed in this study were sourced from the TriNetX Research Network (https://trinetx.com). Access to this database is available to registered institutions through a subscription agreement. No new data were created, and raw data cannot be shared publicly due to data use agreements and privacy restrictions.

## Abbreviations

The following abbreviations are used in this manuscript:

THA	Total Hip Arthroplasty
rTHA	Revision Total Hip Arthroplasty
SSI	Surgical Site Infection
PJI	Periprosthetic Joint Infection
